# Research Progress of Metabolomics in Asthma

**DOI:** 10.3390/metabo11090567

**Published:** 2021-08-24

**Authors:** Chao Wang, Shengyu Jiang, Siyu Zhang, Zhuoer Ouyang, Guoqiang Wang, Fang Wang

**Affiliations:** 1 Department of Pathogen Biology, College of Basic Medical Sciences, Jilin University, Changchun 130021, China; wangc19@mails.jlu.edu.cn (C.W.); jsy20@mails.jiu.edu.cn (S.J.); zsy20@mails.jlu.edu.cn (S.Z.); 2 Department of Cellular Biology, College of Basic Medical Sciences, Jilin University, Changchun 130021, China; oyze20@mails.jlu.edu.cn

**Keywords:** metabolomics, asthma, inflammation, pathogenesis

## Abstract

Asthma is a highly heterogeneous disease, but the pathogenesis of asthma is still unclear. It is well known that the airway inflammatory immune response is the pathological basis of asthma. Metabolomics is a systems biology method to analyze the difference of low molecular weight metabolites (<1.5 kDa) and explore the relationship between metabolic small molecules and pathophysiological changes of the organisms. The functional interdependence between immune response and metabolic regulation is one of the cores of the body’s steady-state regulation, and its dysfunction will lead to a series of metabolic disorders. The signal transduction effect of specific metabolites may affect the occurrence of the airway inflammatory immune response, which may be closely related to the pathogenesis of asthma. Emerging metabolomic analysis may provide insights into the pathogenesis and diagnosis of asthma. The review aims to analyze the changes of metabolites in blood/serum/plasma, urine, lung tissue, and exhaled breath condensate (EBC) samples, and further reveals the potential pathogenesis of asthma according to the disordered metabolic pathways.

## 1. Introduction

Asthma is a highly heterogeneous disease characterized by an inflammatory response in the airways. With nearly 300 million people in the world suffering from asthma, precise and personalized treatment of asthma patients is extremely important [1]. Some of the current traditional clinical tests for asthma, such as quantitative sputum cytometry, blood eosinophil count, a fraction of exhaled nitric oxide (FeNO), and serum IgE, are almost impossible to determine in early asthma [2]. Asthma is a complex disease composed of different endotypes with different inflammatory and clinicopathological characteristics, so the clinical phenotype of asthma is extremely complex. It is difficult to make accurate judgments in clinical diagnosis and identification, so it is almost impossible to provide precise and personalized treatment for asthma patients [1,3]. Metabolomics, as an emerging method of research, can better reflect the phenotypes of complex diseases and their pathophysiological changes, and to some extent, even elucidate the pathogenesis of diseases from a metabolic perspective. In a highly heterogeneous and severely phenotypically complex disease such as asthma, metabolomic study tools from studies of large clinical cohorts may become a better research method to obtain more comprehensive information than simple cytometric metrics [4,5]. Comprehensive understanding of asthma-related metabolomic data can provide powerful clues or evidence for precise and personalized diagnosis, treatment, and prognosis of asthma patients, which can further help clinical implementation of personalized patient treatment plans [4]. In addition, differential metabolites or called biomarkers screened by a large amount of metabolomics data are likely to be one of the most important pieces of evidence to explain the mechanism of asthma pathogenesis. In this review, recent metabolomic studies on asthma were integrated and analyzed in different categorical samples, and the potential contributions of certain metabolites and metabolic pathways to the phenotypic classification, treatment, and diagnosis of asthma were discussed and summarized in order to further elaborate the possible pathogenesis of asthma from a metabolic perspective. We expect that this review will provide a complete report on asthma and related biomarkers, integrate and analyze the progress of asthma metabolomics for the reader, and provide a basis for further studies to explore the pathogenesis of asthma.

## 2. Integrative Analysis of Asthma-Related Metabolites and Metabolic Pathways in Different Samples

**Figure 1:**
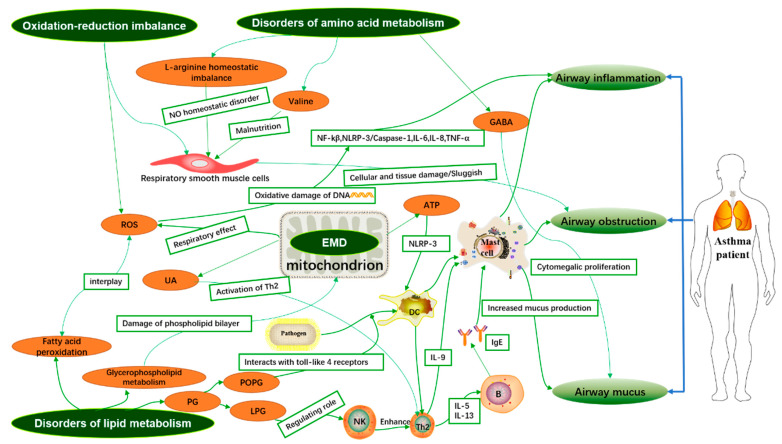
Mechanism of asthma based on metabolites. Disturbances in lipid metabolism, amino acid metabolism, energy metabolism, and oxidative–oxidative imbalance lead to inflammation and oxidative stress, which may be involved in the development of asthma. GABA: gamma-aminobutyric acid; NF-κB: Enhanced κ-light chain in nuclear factor-activated B cells. NLRP-3: nod-like receptor family pyrin domain containing 3; IL-6: interleukin-6; IL-8: interleukin-8; IL-5: interleukin-5; IL-13: interleukin-13; IL-9: interleukin-9; IgE: Immunoglobulin E; TNF-α: Tumor Necrosis Factor α; ROS: reactive oxygen species; NK: natural killer cell; Th2: T helper 2 cell.

High-throughput sequencing technology has been widely applied to the phenotypic identification, diagnosis, and intervention of highly heterogeneous and complex diseases such as asthma. The metabolomic analysis of various samples. i.e., blood serum plasma samples, urine samples, local tissue samples, exhaled breath condensate (EBC) samples, bronchoalveolar lavage fluid (BALF) samples, induced sputum, and stool samples showed that the different metabolites associated with asthma in different samples and the integration of disordered metabolic pathways may provide evidence for the pathogenesis of asthma. This review explores the potential association of different asthma metabolites in various samples with airway inflammation, airway obstruction, and mucus secretion during the development of asthma, and explains the possible pathogenesis of asthma from a metabolic perspective as shown in Figure 1.

The keywords “asthma” and “metabolomics” were searched in PubMed, and the following criteria were used for screening: (1) clinical studies or cohort studies with human samples in the last 5 years; and (2) studies must include asthma patients and normal controls. We obtained a total of 19 references, of which 9 studies were blood/serum/plasma samples, 6 studies were urine samples, 1 study was cellular samples, 2 studies were EBC samples, and 1 study was sputum samples. In addition to this, one clinical control study of BALF samples was manually searched and added. We screened 20 research papers on asthma metabolomics and integrated the asthma-related differential metabolites into Table 1, which can more visually present the asthma metabolomics research in recent years and provide readers with a comprehensive report on asthma metabolomics analysis. Apart from that, the biomarkers in Table 1 are likely to be one of the significant evidence to explain the pathogenesis of asthma, and their disordered metabolic pathways after integration may, to some extent, better explain the pathogenesis of asthma from a metabolic perspective.

### 2.1. Analysis of Biomarkers Associated with Asthma in Blood/Serum/Plasma Samples

Metabolomics of blood/serum/plasma samples reflects changes in organic global metabolites. The levels of metabolites in blood/serum/plasma may change when the organism has a local disease or dysregulation. Metabolomic studies of blood serum plasma samples clearly demonstrate that the body’s stress response to this local disease or local dysfunction is reflected by the level of metabolites in the sample. Metabolomic studies of blood/serum/plasma provide a more comprehensive picture of global changes in the body system and help us to explore the pathogenesis of asthma disease from a metabolic perspective.

#### 2.1.1. Phenotypic Identification and Treatment of Asthma

Traditional asthma-phenotyping criteria are based on a set of features and clinical characteristics that are considered to be a syndrome rather than a particular disease diagnosis, such as proportion of eosinophils in sputum, obesity, age, and presence of severe airflow obstruction with bronchodilator responsiveness [26]. Researchers have proposed a classification of inflammatory phenotypes of asthma based on the characteristics of airway inflammatory cells: the eosinophilic asthma and non-eosinophilic asthma [27]. Neutrophilic asthma, also known as steroid-resistant asthma, is not sensitive to glucocorticoid therapy, and the clinical treatment options for asthma patients with different inflammatory phenotypes are also different.

The mechanisms of airway inflammation in asthmatics are complicated, involving various types of cells and a large number of metabolic pathways, and the various metabolites produced by cells involved in metabolic processes may play a major role in the pathogenesis of asthma. Most asthmatic patients suffer from type 2 inflammation, which is associated with certain cytokine profiles (IL-4, IL-5 and IL-13) and inflammatory cells (eosinophils, mast cells, basophils, type 2 T helper lymphocytes, and immunoglobulin E producing plasma cells) [28]. The release of cytokines from epithelial cells, especially interleukin-33 (IL-33), induces the expression of OX 40 ligand on dendritic cells (DCs), which in turn activates the migration of primitive CD4+ T cells to the B-cell area and promotes the maturation of Th2 cells [29]. Th2 cells migrate to the airway epithelium, and cells of the epithelial mucosa secrete IL-5 and IL-13 and mediate changes in airway mucosal inflammation and remodeling [28,29]. In addition, bronchial smooth muscle contraction causes airway narrowing. Smooth muscle also causes bronchial inflammation by secreting a series of inflammatory mediators that recruit and activate inflammatory cells, such as mast cells or T lymphocytes [30].

Serum glycerophospholipid metabolic profile is significantly different between eosinophilic and non-eosinophilic asthmatic patients [31]. The levels of Lysophosphatidylglycerol (LPG) were significantly elevated in the metabolic profile of patients with eosinophilic asthma, and LPG may be an important biomarker for asthma typing [32]. The metabolomics can be further used to effectively classify asthma patients according to the level of glycerophospholipids, which can be used for subsequent clinical treatment. There are different metabolic profiles for various clinical inflammatory phenotypes of asthma in northeastern China. The findings of the serum metabolic profile showed significant changes in three metabolic pathways: glycerophospholipid, retinol, and sphingolipid metabolism [6]. The metabolic physiological activities of glycerophospholipids, phospholipids, and retinol are closely related to the pathogenesis of the inflammatory phenotype of asthma. Serum metabolic profile also differs in patients with mild, moderate, and severe asthma, with oleic acid ethanolamine increasing with asthma severity [33]. Unconjugated bilirubin was strongly associated with childhood asthma and recurrent wheezing in early childhood [9]. The metabolomic signature of childhood patients with co-existing food allergies and asthma was also markedly altered in lipid metabolites [34], most notably sphingolipids and ceramides. There was evidence that dust mite sensitization in asthmatic children is associated with microbial carbohydrate, amino acid, and lipid metabolism [35]. Clinical mild, moderate, and severe asthma substyles can be distinguished by plasma metabolic analysis of patients. Of even greater concern is the fact that worsening asthma is associated with severe childhood morbidity and mortality. Repeated asthma attacks can lead to the progressive loss of lung function, which is sometimes fatal or near fatal [36]. In recent decades, there has been increasing evidence that metabolic changes are associated with immune inflammation and clinical outcomes in obese asthma [37,38,39,40]. The metabolic characteristics of obese asthma are different from those of lean asthma, and the metabolic spectrum of serum showed that the contents of valine, uric acid, and N-methyl-DL-alanine β-glycerophosphate in serum of obese patients with asthma were higher than those of patients with lean asthma, while the contents of asparagine 1 and D-glyceric acid were decreased [41]. Furthermore, a recent study suggested that the relative proportion of acetic acid in obese children with asthma was significantly lower than in children with normal weight asthma [42]. The difference in the pathway of bioenergy metabolism between thin and obese asthmatic patients is partly due to the different sensory effects of NO signals [43]. In summary, the potential metabolic characteristics of these different phenotypic asthma reveal their immune metabolic mechanism, and metabolomics can be used to help the clinical phenotypic diagnosis and treatment of asthma.

Currently, short-acting beta agonists (e.g., salbutamol) are the most frequently used drugs for the medical treatment of asthma [44]. The serum metabolome revealed that sustained albuterol β2 receptor activation in normal healthy subjects promoted lactate production and altered aerobic glycolysis, gluconeogenesis, and free fatty acid production, whereas arachidonic acid metabolism and linoleic acid metabolic pathways were altered during asthma control with albuterol, and two metabolites -monoHETE_0863 and sphingosine-1-phosphate (S1P) were significantly modified before and after asthma control [45]. S1P is a potent leukocyte chemokine that organizes the migration of lymphocytes and is involved in several major symptoms of asthma, such as airway hyper-reactivity and pulmonary eosinophil sequestration [46,47], and S1P has been identified as a possible drug target for the treatment of asthma. The above studies suggested that lipid mediators played an essential role in airway inflammation, and the sphingolipid metabolites were new molecular candidates for future functional validation studies. In clinical trial research, levels of dehydroepiandrosterone sulfate, cortisone, cortisol, prolylhydroxyproline, pipecolate, and N-palmitoyl taurine were found to be significantly correlated with inhaled doses of glucocorticoids [33].

In recent years, traditional Chinese medicine and its prescriptions have attracted enormous interest for their low side effects in asthma treatment. Modified Kushen Gancao Formula (MKG) extracted from traditional Chinese medicine exerted beneficial therapeutic effects on experimental allergic asthma by regulating the disorders of fatty acid metabolism, sphingolipid metabolism, glycerophospholipid metabolism, and arachidonic acid metabolism [48]. After the treatment of asthma of rats with dry ginger and Linggan Wuwei Jiangxin decoction, most of the metabolites and metabolic pathways disrupted by asthma can be restored to normal levels [49]. Gu-Ben-Fang-Xiao decoction regulates the protein kinase (AMPK) pathway to regulate fatty acid metabolism and thereby alleviate asthma [50]. The main metabolic change we observed was an altered glycerophospholipid metabolic pathway in patients with asthma [12,13,33]. In addition, Rhodiola wallichiana var. cholaensis (RWC) significantly improved steroid resistance in neutrophilic asthma [51]. The combination of RWC and dexamethasone treatment in asthma model mice affected the metabolic profile of the asthma phenotype [52], and the combination of the two drugs treated steroid-resistant asthma through significant modulation of linoleic acid metabolism, glycerophospholipid metabolism, and primary bile acid biosynthetic metabolism, which was significant for modern clinical drug use research.

The serum metabolic profile of the animal model of OVA-induced asthma is distinct from that of normal controls [53], and this change is also mainly a disturbance of the glycerophospholipid metabolic pathway, which is very similar to that of clinical patients. However, the major metabolic disorder in asthma models can be reversed by surfactant protein A (SPA), and, from the metabolic point of view, SPA is highly likely to improve the condition of asthma patients and may be a potential drug for the treatment of asthma [53]. 12-OH-17,18-Epoxyeicosatetraenoic acid ameliorates eosinophilic airway inflammation in mice and is a potential target for the treatment of asthma [54].

#### 2.1.2. Diagnosis of Asthma

We can distinguish asthmatic patients from healthy individuals based on their different plasma metabolomic profiles, and plasma metabolomic analysis of asthmatic patients can, to some extent, point to the activation pathways of their inflammatory and immune pathways [55]. Serum sphingolipid metabolism was significantly different between asthmatics and healthy controls, with plasma sphingomyelin (SM) levels being significantly lower in asthmatics than in healthy controls [7], and this study suggested that SM may be a protective factor in asthma and may be involved in the pathogenesis of asthma. SM is also a human CD300f physiological receptor ligand that inhibits receptor-mediated activation of high-affinity IgE mast cells [56]. Plasma histidine levels were significantly higher in children with asthma than in normal children [8,57], and there were significant alterations in lipid metabolic pathways and purine metabolic pathways [58]. In contrast, the metabolomic profile of childhood asthma with airway hyperresponsiveness was distinctly different compared to other types of asthma, with associated metabolites being polar and nonpolar lipids [59], suggesting a change in the lipid composition of plasma in childhood asthma patients.

Serum metabolomics results suggested that lipid metabolism in asthmatic patients was significantly different from that in healthy subjects, with a significantly altered glycerophospholipid metabolite profiles in the plasma of adult asthmatics [60]. Furthermore, abnormal lipid metabolism is associated with severity and IgE levels in asthmatics [10]. It is obvious to show that asthma attacks are closely associated with related lipid metabolites which may have a strong correlation with the diagnosis, treatment, and prognosis of asthma. Long chain-free fatty acids (LCFFAs), a large class of biomarkers for asthma [11], play significant roles in physiological activities such as inflammation occurrence, tissue repair, and immune cell behavior [61], which may be closely involved in the airway inflammatory response in asthma.

However, the degree of bronchodilator response (BDR) in treated asthmatic patients was different at each age level, and the age level of asthmatic patients was negatively correlated with BDR [62,63]. In addition, the serum metabolomic of asthmatic patients suggest that the increased levels of cholesterol ester, gammaminobutyric acid, and ribothymidine may attenuate the age-associated BDR decline [64]. In this follow-up study, phosphatidylethanolamine (PE) and sphingomyelin (SM) metabolites were identified to be associated with circulating fibrinolytic enzymes [65]. Fibrinolytic enzymes may alter the structural properties of lung surfactant and thus affect BDR. In further studies on lipid metabolism associated with asthma, there have also been correlations between nicotinamide and pyrimidine metabolism, bile salt production, heme catabolism, and microbial-related secondary metabolism, and the occurrence of asthma [66].

A serum amino acid metabolomics study in asthmatics showed that serum levels of taurine, l-valine, and DL-β-aminoisobutyric acid were lower in asthmatics than in healthy controls, while levels of ƴ-amino-n-butyric acid and l-arginine were higher in asthmatics than in controls, and plasma tryptophan levels were elevated in asthmatics [12,67,68]. Amino acids exert antioxidant and immune activities related to asthma pathogenesis through metabolic activities [69], suggesting that amino acid metabolism also plays an important role in the development and progression of asthma.

Asthma and chronic obstructive pulmonary disease (COPD) have similar clinical manifestations and pulmonary function diagnostics, and the diagnosis of these two diseases can sometimes be difficult to differentiate in the clinical setting [70]. Moreover, asthma and COPD are both highly heterogeneous diseases, and their respective mechanisms are extremely complex, which can cause some problems in the diagnosis and treatment of asthma. Serum metabolomics can reveal asthma and COPD communalities, as some energy metabolites, cholesterol, and fatty acids are significantly associated with immune mediators [13]. However, the differences are that serum levels of linoleic acid, hypoxanthine, l-pipecolic acid, p-chlorophenylalanine, and acetylcarnitine are significantly higher in asthma patients than in COPD patients, while levels of metabolites, such as α-N-phenylacetyl-l-glutamine, 1-methyladenosine, glycochenodeoxycholate, l-citrulline, and l-glutamine, are significantly lower than in COPD patients [14]. There was also evidence that alterations in purine metabolic pathways may be associated with the development of asthma [71], and serum metabolomics can well differentiate asthma from COPD patients and help improve the clinical diagnosis of asthma.

### 2.2. Analysis of Biomarkers Associated with Asthma in Urine Samples

Urine sample is also one of the easily obtained and commonly used samples in the study of metabolomics, because the metabolic components of urine change early and to a great extent in the occurrence and development of the disease, so it can reflect the pathophysiological state of systemic or local injury. Urine collection is non-invasive, simple, and convenient, and is very suitable for sampling and collection of children. However, the limiting factors of urine sample are that body water intake, physiological factors, and other external factors will affect the voiding volume, which will cause irregular fluctuations in the concentration of metabolites as well as some interference to the follow-up analysis.

1-methylnicotinamide and trimethylamine N-oxide (TMAO) were significantly lower in urine samples from children with asthma than in normal children [38,41,61]. Dimethylamine played an important role in the development of asthma, and three metabolites (1-methylnicotinamide, allantoin, and guanidoacetic acid) were significantly correlated with asthma [15]. There was evidence of disturbance of three major metabolic pathways in the urine of children with asthma: sphingolipid metabolism, protein biosynthesis, and citric acid cycle [16]. In contrast, children with the transient wheezing phenotype showed different urinary metabolites compared to children with early-onset asthma [18], and were also able to be distinguished from healthy children. Interestingly, the metabolic profile of urinary organic acids was significantly different between the sexes of children with asthma, which may be related to hormonal changes and environmental exposure differences in children of different sexes [72,73]. Bile acid taurochenodeoxycholate-3-sulfate, fatty acid 3-hydroxytetradecanedioic acid, and a glucoronidated steroid compound may be differential metabolites of asthma in children [17]. Eight kinds of urine metabolites (aspartic acid, stearic acid, heptadecanoic acid, threitol, acetylgalactosamine, xanthosine, hypoxanthine, and uric acid) have a good ability to distinguish asthma [19].

Urine metabolomics can also be used as a feature to distinguish and diagnose asthmatics from COPD patients, and although there is no significant difference between the urine metabolites of asthmatics and COPD patients after rectification, overall metabolite levels are lower in COPD patients than in asthmatics [20]. The diagnostic model constructed by differential metabolites of non-targeted metabolites in urine can accurately distinguish asthma patients from COPD patients in a small sample range [74]. The investigation of therapeutic strategies for asthma, especially for steroid-resistant asthma, has been a major concern in respiratory medicine. It has indicated that tyrosine metabolism, degradation of aromatic compounds, and glutathione metabolism may be important pathways in glucocorticoid-resistant asthma [75]. Moreover, glutathione and γ-glutamylcysteine may become important drug targets. Unlike serum metabolomics, urine metabolomics focuses on organic acid metabolites and would better complement the pathophysiological mechanisms underlying the onset and development of asthma.

### 2.3. Analysis of Biomarkers Associated with Asthma in Lung Cell and Tissue Samples

The metabolomics study of local tissue or cell samples can better show the local metabolic physiological activities of the body, and can more accurately show the occurrence and development of the disease from the level of cell metabolism. The biomarkers of bronchial epithelial cells (BECs) in patients with severe asthma and healthy people are mainly concentrated in purine metabolism, amino acid biosynthesis, and glycolysis [21]. Another eosinophil metabonomics study showed a decrease in Protectin D1 biosynthesis [76], which may be highly associated with asthma inflammation. Metabolomic approaches to polyamines of lung tissue of mice with asthma showed that the overall level of polyamines in the asthma group was significantly higher than that in the control group, and putrescine and N^1^-acetylputrescine were important biomarkers of asthma [77]. Aspartic acid, butanoic acid, glucose, hexadecanoic acid, idose, inosine, isoleucine, malic acid, octadecanoic acid, serine, uric acid, and valine can be used as biomarkers in lung tissue of asthma caused by PM2.5 [78].

### 2.4. Analysis of Biomarkers Associated with Asthma in EBC

In addition to the existing clinical methods for diagnosis, phenotypic identification, and monitoring of asthma, breathomics is a very practical non-invasive method [79]. EBC metabolomics has shown good predictive accuracy for the diagnosis of asthma, but the clinical sample is still insufficient, thus limiting the clinical application of breathomics in the early diagnosis of asthma [80]. Although breathomics is still in the exploratory stage, this non-invasive method is likely to be a useful and practical tool for the diagnosis, phenotype identification, monitoring, and personalized clinical treatment of childhood asthma in the future.

Amino acid lysine levels in EBC samples from asthma patients was the only metabolite that could differentiate severe from mild-to-moderate asthma [81]. Recent work has shown that EBC metabolomics can distinguish asthmatics from healthy individuals and can subdivide subtypes of severe asthma [82,83], which has important implications for clinical phenotypic diagnosis and treatment. Another clinical trial showed that the relative abundance of four metabolites, i.e., lactate, formate, butyrate, and isobutyrate, were significantly higher in EBCs of children with asthma than in healthy controls [22]. Three metabolites (prostaglandins, fatty acids, and glycerol phospholipids) can be used as biomarkers of breathomics to distinguish asthma children from healthy children [23]. Obesity and asthma are closely linked, and, interestingly, the EBC metabolomics of obese asthmatics is characterized by a unique metabolic spectrum compared to obesity or asthma alone [84]. Similar to the serum OR urine metabolomics [14,20], NMR metabolic spectra of EBC can be used to distinguish asthmatics from COPD patients, and, interestingly, smoking may not be a major factor in the metabolic spectrum differentiation between asthma and COPD [85].

### 2.5. Analysis of Biomarkers Associated with Asthma in Other Samples

Bronchoalveolar lavage fluid (BALF) and induced sputum samples are valuable in the diagnosis and study of pulmonary respiratory diseases, such as COPD and asthma [86]. The BALF and induced sputum samples can be used to provide insight into the status of various diseases, including, of course, the pathogenesis of asthma [87]. BALF and induced sputum are often better than systemic biological fluids (e.g., plasma) because they are nearest to the site of disease and can reflect the local pathophysiological state of the organism. However, the application of such invasive sampling methods setting is their limitation in the clinical. Lipids are important components of lung tissue surfactant, and several previous studies have found that lipids play an essential role in the development and progression of asthma [16,33,39,44]. Although the exact mechanism is still unknown, quite similar to the results of blood metabolomics, one study showed a significant increase in lipid levels in BALF of asthmatic patients [24]. Although the exact mechanism is still unclear, similar to the results of blood metabolomics, it also showed a significant increase in lipid levels in BALF of asthma patients, suggesting that both metabolomic analysis of systemic body fluids and metabolomic analysis of local body fluids can reveal local changes in metabolites of damage or illness in the body, but their respective focuses may be different. Metabolites in the sputum of asthmatics and healthy people are significantly different [88]. Another investigation showed that sputum metabolomics were able to distinguish between asthmatics and healthy individuals, integrating pathways involving glycerophospholipid metabolism, inositol phosphate metabolism, glycolysis, or gluconeogenic metabolic pathways [25].

In addition, the occurrence and development of asthma may be associated with gut microbial metabolism [89,90,91], and metabolic disturbances in the gut may be strongly related to some secondary metabolites of gut microbes such as β-alanine, 4-hydroxybutyrate, and butyrate [90]. In one study, Gu-Ben-Fang-Xiao decoction was found to further improve asthma in mice by modulating the metabolic activity of intestinal microorganisms [91].

## 3. The Potential Relationship between Disorders of Metabolites and Metabolic Pathways in the Organism and the Pathogenesis of Asthma

The pathogenesis of asthma is extremely complex and is influenced by various factors, but the ultimate manifestation of asthma is the airway inflammatory response, which is inseparable from the inflammatory immune mechanisms of the organism. Atypical metabolic reprogramming caused by extrinsic factors, such as allergens, viruses, contaminants, diet, or microorganisms, may lead to cellular metabolic dysfunction and defective immune responses in allergic diseases [92].

Metabolomic analysis of the development of asthma may provide more comprehensive information. We used the differential metabolites and disordered metabolic pathways in asthma to further elucidate the inflammatory immune mechanisms of asthma development (Figure 1).

The studies we have integrated include metabolomic studies on asthma in different types of samples (blood/serum/plasma samples, urine samples, EBC, BALF, and sputum samples). Metabolomic studies of different types of samples will focus on different types of metabolites with metabolite levels in blood/serum/plasma samples and urine samples reflecting systemic metabolic disturbances in lung diseases such as asthma, while EBC, BALF, and sputum samples focus on local metabolic disturbances in asthma. We believe that the integrated analysis of metabolites in these different samples will be more comprehensive and reasonable than the metabolomic analysis of a single sample, and that these studies are the most recent ones in recent years. With the advancement of technology and the expansion and enrichment of metabolite databases, studies on asthma metabolomics 5 years ago may be far less accurate or comprehensive than the current metabolomic studies. However, the metabolomic studies on asthma in this review involve metabolomic studies of asthma patients from different national regions, different ethnic groups, and different ages. Furthermore, the back-ground levels of metabolites are different, for example, age factors can influence the levels of metabolites in cohort studies. Certainly, in general, it is reasonable and feasible for the study to perform an integrative analysis.

### 3.1. Lipid Metabolism and Asthma

The disorders of lipid metabolism in asthma patients mainly involve glycerophospholipid metabolism and fatty acid metabolism. Moreover, glycerophospholipids are the most abundant phospholipids in the organism, and their dominant glycerophospholipid metabolism is intimately related to the occurrence of asthma. Phosphatidylglycerol (POPG) composed of phosphatidylglycerol (PG) is an important component of human lung surfactant, and POPG can inhibit lipopolysaccharide-induced pro-inflammatory cytokine production by directly interacting with CD 14 and MD-2 on Toll-like receptor 4 [93,94]. In the case of asthma, however, the LPG produced by the hydrolysis of PG may be able to enhance the Th2 level in asthma by regulating the chemotaxis of NK cells [95], inducing the accumulation of eosinophil infiltration and causing an inflammatory response [96]. SM may be involved in asthma pathogenesis, and it may be a protective factor in asthma [7]. A decrease in sphingolipid synthesis can induce the development of airway hyperreactivity, which may be related to the contraction of airway smooth muscle [97,98]. Lipid inflammatory mediators are metabolites that are derived from omega-6 (n-6) or omega-3 (n-3) fatty acids; n-3 polyunsaturated fatty acids (PUFA) or monounsaturated fatty acids (MUFA), which reduce inflammation; and saturated fatty acids (SFAs). which may be involved in pro-inflammatory responses [99]. Prenatal intake of olive oil affects immunomodulatory genes of placental histone acetylation, confirming the pro-acetylation effect of olive oil polyphenols [100,101]. Polyunsaturated fatty acids can reduce asthma inflammation by inhibiting the arachidonic acid metabolism and reducing the production of both leukotrienes and prostaglandins [102]. Short-chain fatty acids (SCFAs), such as butyric acid, produced by fermentation of dietary fiber by intestinal microorganisms, can inhibit pulmonary function of II innate lymphoid cells (ILC 2) and the subsequent development of airway hyperresponsiveness (AHR) [103]. Arachidonic acid metabolites may be a biomarker of the occurrence and treatment of allergic asthma, and can also be used as a new biological target for monitoring the therapeutic effect of subcutaneous immunotherapy (SCIT) [104].

Whereas oxidative stress has a significant impact in the pathophysiology of asthma [105]. Researches have suggested that activated and recruited inflammatory cells in asthma trigger respiratory storms, which in turn produce large amounts of ROS and subsequently lead to tissue damage in the body [106,107]. Other studies suggested that exogenous ROS would activate the downstream NF-kβ inflammatory pathway through a series of cellular signaling pathways and increase the transcriptional levels of promotional genes, leading to the persistence of inflammation and enhancing the inflammatory process [108,109,110]. Fatty acid oxidation (FAO) is enhanced in asthmatics [111], and inhibition of the FAO process attenuates AHR and inflammatory immune cell recruitment and reduces the production of allergen-specific IgE and asthma-associated cytokines and chemokines [112], which may provide a novel therapeutic approach for the treatment of asthma.

### 3.2. Amino Acid Metabolism and Asthma

Amino acids as anti-oxidant are immunologically active mediators of asthma [12], and amino acid metabolism may be implicated in the pathogenesis of asthma. Valine, an essential branched-chain amino acid, performs an important role in muscle metabolism, and low amounts of valine in asthmatic patients may be correlated with malnutrition of respiratory smooth muscle, making smooth muscle expansion retarded [113]. High levels of gamma-aminobutyric acid (GABA) in asthmatics may be connected with cupric cell proliferation and excessive secretion of mucus [114].

Intriguingly, the variation of arginine levels in adult asthma and childhood asthma may not be identical [12,69,115], and perhaps there might be a possibility that the level of l-arginine is linked to the severity of asthma in patients [116]. Levels of l-arginine in mild asthmatics are consistent with those in normal subjects and may differ from those in severe asthmatics, but of course these are speculations based on existing studies and need to be verified in further large-scale clinical studies. Nevertheless, existing studies have suggested that l-arginine/NO steady state may be directly tied to the pathogenesis of asthma [117] and that alterations in l-arginine homeostasis may promote asthma by reducing nitric oxide (NO) production and increasing the formation of peroxynitrite, polyamines, and l-proline [118]. Furthermore, lowering l-arginine levels caused oxidative stress, nitrosative stress, and bronchial hyperreactivity in the absence of any airway inflammation [119,120]. In conclusion, arginine has a significant role in the pathogenesis of asthma. Microbial secondary metabolic derivatives of l-tyrosine are protective against allergic airway inflammation, perhaps associated with the decoupling of Toll-like receptor 4 (TLR 4) signaling in airway epithelial cells [121]. In contrast, the tryptophan metabolic pathway and its production (5-hydroxytryptamine) play an instrumental function in allergic reactions [122], perhaps in relation to the development of allergic airway inflammation (including, of course, allergic asthma).

### 3.3. EMD and Asthma

Energy metabolism disorders (EMD) might cause an increase in ROS and a decrease in airways epithelial cell function, which could aggravate the onset of asthma [123]. Uric acid (UA), the eventual oxidation product of purine metabolism, acts as an initiator and magnifier of Th2 cell immunity and allergic inflammatory responses in the mouse by activating inflammatory endritic cells (DCs) [71]. UA released in vivo promotes Th2 cell activation by promoting the production of innate prospective Th2 cytokines that instruct DCs to induce Th2 cell immunity [124]. UA released upon alum injection in vivo induces Th2 cell adjuvant effects that do not depend on the inflammasome-IL-1R pathway [71]. UA homeostasis is associated with the onset of inflammation, and the UA at lower concentrations can handle oxidative stress induced by the external environment and maintain intra-tissue homeostasis, while at higher concentrations it can induce an immune response in the respiratory mucosa of the body [125]. Air pollution, allergens, and viral infections may lead to the increase in uric acid levels [126,127], while the inflammatory response may be associated with Th2 immune response and ILC2 accumulation [71,128]. Furthermore, when some external stimuli, such as bacterial LPS and yeast polysaccharides, increase the process of glycolysis, which in turn activates DCs, resulting in the secretion of IL5, IL9, and IL13 by Th2 cells [129,130], an increase in eosinophils and IgE production by B cells, inducing mucus production and airway remodeling. Regulation of the disruption of energy metabolism in mitochondria may be a new approach to the treatment of asthma [131]. Accordingly, the accumulation of uric acid activates eosinophils to autocrine ATP which is known to be a core element in energy metabolism and an activator of the NLRP 3 inflammasome [132], leading to the release of IL-1β, and also exerts an influential impact in the inflammation of pulmonary asthma through purinergic signaling [132,133,134]. 

### 3.4. Oxidation-Reduction Imbalance and Asthma

Oxidative stress plays a crucial role in airway inflammation, such as bronchial asthma, and patients with acute asthma attacks display increased oxidative stress [135,136]. Correspondingly, cytotoxic activity of lung tissue in smoking asthmatics is enhanced with oxidative DNA damage [137]. Mitochondrial respiration is the main source of ROS, and large amounts of ROS trigger the release of proinflammatory cytokines and activate inflammatory signaling pathways [138,139,140,141]. In contrast, oxidative stress in the adipose tissue of obese asthmatics may be linked to the genesis of their asthma, and lipid peroxidation metabolites of linoleic acid and lipoxygenase may also contribute to respiratory epithelial damage [123,142]. The dysregulation of oxidation-reduction homeostasis in pathological states leads to excessive production of ROS, resulting in oxidative stress and oxidative damage to cells or tissues [143]. In the lungs, this manifests itself in the form of oxidative damage to respiratory epithelial cells and tissues, which recruits the aggregation of immune cells, causing inflammatory infiltration and triggering inflammatory features. Of course, the relationship between the occurrence of inflammation and the redox homeostasis of the body is extremely complex and interacts with each other [143]. The expression and activation of the epidermal growth factor receptor (EGFR) is significantly enhanced in the airways of asthmatic patients, and EGFR stimulates the secretion of mucin and IL-8 by respiratory epithelial cells, thereby producing airway mucus and causing airway remodeling [144,145]. Both oxidative deactivation of protein tyrosine phosphatases and cysteine oxidation of the EGFR structural domain activate and enhance EGFR signaling [146], thereby exacerbating the course of asthma. Oxidative stress may also impair TLR 3 receptors in bronchial epithelial cells of asthmatic patients, with reduced antiviral signaling and impaired antiviral responses, leading to more airway inflammation [147]. Repairers of oxidative DNA damage mediate the secretion of mediators of bronchoconstriction by mast cells and smooth muscle cells [140].

## 4. Conclusions

The metabolic substances and disordered metabolic pathways involved in the pathogenesis and development of asthma are interrelated. Lipid metabolism, amino acid metabolism, nucleotide metabolism, carbohydrate metabolism, and other metabolic pathways are involved in energy metabolism. These metabolic pathways involve metabolites that are more or less related to energy changes. Disturbed energy metabolites and imbalance of oxidation-reduction homeostasis in the organism can elucidate the pathogenesis of asthma from a metabolic standpoint. The disordered metabolic pathways associated with asthma affect airway inflammation, oxidative stress, and airway remodeling. Perhaps the application of metabolomics will better explain the occurrence and development of highly complex diseases, such as asthma, in the future.

However, there are limitations in explaining the pathogenesis of asthma by a single metabolomics. In contrast, the recent emergence of combined multi-omics studies from different perspectives of genomics, transcriptomics, proteomics, epigenomics, microbiomics, and metabolomics can provide a better understanding to explain the pathogenesis of asthma, a highly heterogeneous and complex disease, which can be of major value for the future diagnosis, phenotypic discrimination, treatment, and prognostic observation of asthma [148]. The application of metabolomics in combination with other histological techniques can laterally validate the results of metabolomics and better explain the function played by metabolites in diseases. Combined multi-omics study analysis can further improve the diagnosis of asthma, refine the phenotypic classification of asthma, and personalize effective treatment of asthma-related respiratory diseases.

## Figures and Tables

**Table 1:** A summary of asthma-associated metabolomic studies.

Author and Year	Subjects	Sample/Methods	Significant Metabolites	
			Up	Down
Pang, Z. et al. (2018) [6]	eosinophilic asthmatics (EA, *n* = 13), noneosinophilic asthmatics (NEA, *n* = 16), and healthy controls (HC, *n* = 15)	Serum/UPLC-MS/MS	Monosaccharides, LysoPC(18:1), Retinyl ester, PC(18:1/2:0), LysoPC(o-18:0), Arachidonic acid, PE(18:3/14:0), PC(16:0/18:1)	Glycerophosphocholine, PS(18:0/22:5), Cholesterol glucuronide, Phytosphingosine, Sphinganine, LysoPC(p-18:1), Retinols, PC(20:4/16:1)
Guo, C. et al. (2021) [7]	51 asthma patients and 9 healthy individuals	Serum/LC-MS	No report	SM 34:2, SM 38:1, SM 40:1
Chiu, C.-Y. et al. (2020) [8]	Asthma (*n* = 28) and healthy controls (*n* = 26)	Plasma and urine/NMR	Histidine	1-methylnicotinamide, trimethylamine N-oxide (TMAO)
Turi, K.N. et al. (2021) [9]	600 infants from 3 independent cohorts	Plasma/LC-MS	Succinate, N-(2-furoyl)glycine	Iminodiacetate (IDA)
Jiang, T. et al. (2021) [10]	28 healthy controls and 33 outpatients with asthma	Plasma/LC-MS/MS	Phosphatidylethanolamine (PE) (18:1p/22:6), PE (20:0/18:1), PE (38:1), sphingomyelin (SM) (d18:1/18:1), triglyceride (TG) (16:0/16:0/18:1)	Phosphatidylinositol (PI) (16:0/20:4), TG (17:0/18:1/18:1), phosphatidylglycerol (PG) (44:0), ceramide (Cer) (d16:0/27:2), lysophosphatidylcholine (LPC) (22:4)
Bian, X. et al. (2017) [11]	15 healthy human and 15 asthma patients	Serum/UHPLC-Q-TOF-MS	Ursodeoxycholic acid, Deoxycholic acid, Isodeoxycholic acid, EPA	Palmitic acid, Lauric acid
Matysiak, J. et al. (2020) [12]	asthmatic children (*n* = 13) and the control group (*n* = 17)	Blood/LC-MS/MS	l-Arginine, Β-Alanine, Ƴ-Amino-N-Butyric Acid, l-Histidine, Hydroxy-l-Proline	d,l-Β-Aminoisobutyric Acid, Taurine, l-Tryptophan, l-Valine
Ghosh, N. et al. (2020) [13]	(i) controls = 33 (ii) asthma = 34 (iii) COPD = 30 and (iv) ACO = 35	Serum/GC-MS	2-palmitoylglycerol, cholesterol, serine, threonine, Ethanolamine, Glucose, Stearic acid, Linoleic acid, d-Mannose, Succinic acid	Lactic acid, 2-palmitoylglycerol
Liang, Y. et al. (2019) [14]	A total of 17 patients with mildly persistent asthma, 17 patients with stable COPD, and 15 healthy subjects	Serum/LC-MS	Hypoxanthine, P-chlorophenylalanine, Inosine, Theophylline, Bilirubin, Palmitic acid	l-Glutamine, Glycerophosphocholine, Succinate, Xanthine, Arachidonic Acid, l-Pyroglutamic acid, Indoxyl sulfate, l-Valine, l-Norleucine, l-Leucine, l-Phenylalanine
Chiu, C.-Y. et al. (2018) [15]	Asthma (*n* = 30) and healthy controls (*n* = 30)	Urine/NMR	Guanidoacetic acid	1-methylnicotinamide, allantoin
Li, S. et al. (2020) [16]	Asthmatic children (*n* = 30) and healthy controls (*n* = 30)	Urine/GC-MS	l-allothreonine 1, stearic acid, succinic acid, 2-hydroxybutanoic acid, azelaic acid, gentiobiose 2, tyramine, leucine, d-altrose 1, d-erythrosphingosine 1, citraconic acid 4	Valine, uric acid, methionine 1, 3,4-dihydroxycinnamic acid, purine riboside, malonic acid 1, cysteine, erythrose 1, lactamide 1
Chawes, B.L. et al.(2018) [17]	171 and 161 healthy neonates born from mothers with asthma	Urine/UPLC-MS	bile acid taurochenodeoxycholate-3-sulfate, fatty acid 3-hydroxytetradecanedioic acid	glucoronidated steroid compound
Carraro, S. et al. (2018) [18]	Children for transient wheezing (*n* = 16) and early-onset asthma (*n* = 16)	Urine/UPLC-MS	4-(4-deoxy-α-d-gluc-4-enuronosyl)-d-galacturonate, Glutaric acid, 4-hydroxynonenal, Phosphatidyl glycerol, 3-methyluridine, Steroid O-sulfate, 5-hydroxy-l-tryptophan, 3-indoleacetic acid, Tiglylglycine, Indole, Cytosine, N-acetylputrescine, Indole-3-acetamide, 6-methyladenine, 5-methylcytosine, N-acryloylglycine, Hydroxyphenyllactic acid	Oxoadipic acid, (-)-epinephrine, l-tyrosine, 3-hydroxyhippuric acid, Benzoic acid,3-hydroxy-sebacic acid, Dihydroferulic acid 4-sulfate, p-cresol, Indolelactic acid, N-acetyl-l-phenylalanine, N2-acetyl-ornithine
Tao, J.-L. et al. (2019) [19]	Children for healthy control (*n* = 29), uncontrolled asthma (*n* = 37) or controlled asthma (*n* = 43)	Urine/GC-MS	Aspartic acid, Xanthosine, Hypoxanthine, N-acetylgalactosamine	Stearic acid, Heptadecanoic acid, Uric acid, d-threitol
Adamko, D.J. et al. (2015) [20]	Adults with asthma (*n* = 58) and COPD (*n* = 24)	Urine/NMR	Glutamine, succinate, uracil, pantothenate	Arginine, dimethylamine, 3-Hydroxyisovalerate, betaine, choline, glucose, 1-methylnicotinamide
Ravi, A. et al. (2021) [21]	Healthy controls (*n* = 7) and patients with severe asthma (*n* = 9)	BECs/UPLC-MS	Phosphatidylcholines, lysophosphatidylcholines, lysophosphatidylethanolamines, bis(monoacylglycero)phosphates	No report
Chang-Chien, J. et al.(2020) [22]	stable asthma (*n* = 92) and non-asthmatic controls (*n* = 73)	EBC/NMR	lactate, formate, butyric acid, isobutyrate	No report
Ferraro, V.A. et al. (2020) [23]	asthmatic children (*n* = 26) and healthy children (*n* = 16)	EBC/UPLC-MS	9-amino-nonanoic acid, 12-amino-dodecanoic acid, lactone of PGF-MUM, N-linoleoyl taurine, 17-phenoxy trinor PGF2α ethyl amide, lysoPC (18:2(9Z,12Z))	No report
Kang, Y.P. et al. (2014) [24]	38 asthma patients and 13 healthy subjects	BALF/HPLC-QTOF-MS	lysophosphatidylcholine (LPC), phosphatidylcholine (PC), phosphatidylglycerol (PG), phosphatidylserine (PS), sphingomyelin (SM), triglyceride (TG)	No report
Tian, M. et al. (2017) [25]	15 healthy controls and 20 asthma patients	Sputum/UHPLC-QTOF-MS	Glycerol 1-stearate_1, 1-Hexadecanoyl-sn-glycerol_1, Cytidine 2′,3′-cyclic phosphate, 1-Hexadecanoyl-2-(9Z-octadecenoyl)-sn-glycero-3-phospho-(1′-rac-glycerol), 1-Octadecanoyl-2-(9Z-octadecenoyl)-sn-glycero-3-phosphoserine	His-Pro, Thr-Phe_1, Arg-Phe_1, Adenine_1, Phe-Tyr_1, Phe-Gln_1, Tyr-Ala_2, Phe-Ser_1, Urocanic acid

Up: metabolites are higher in asthmatics than in normal people; Down: metabolites are lower in asthmatics than in normal people. UPLC-MS/MS: ultra-performance liquid chromatography-tandem mass spectrometry; LC-MS: liquid chromatography-mass spectrometry; NMR: nuclear magnetic resonance; LC-MS/MS: liquid chromatography-tandem mass spectrometry; GC-MS: Gas chromatography-mass spectrometry; UPLC-MS: ultra-performance liquid chromatography-mass spectrometry; HPLC-QTOF-MS: high performance liquid chromatography tandem quadrupole time-of-flight mass spectrometry; UHPLC-QTOF-MS: ultra-performance liquid chromatography tandem quadrupole time-of-flight mass spectrometry.
